# A Comprehensive, Automatically Updated Fungal ITS Sequence Dataset for Reference-Based Chimera Control in Environmental Sequencing Efforts

**DOI:** 10.1264/jsme2.ME14121

**Published:** 2015-03-19

**Authors:** R. Henrik Nilsson, Leho Tedersoo, Martin Ryberg, Erik Kristiansson, Martin Hartmann, Martin Unterseher, Teresita M. Porter, Johan Bengtsson-Palme, Donald M. Walker, Filipe de Sousa, Hannes Andres Gamper, Ellen Larsson, Karl-Henrik Larsson, Urmas Kõljalg, Robert C. Edgar, Kessy Abarenkov

**Affiliations:** 1 Department of Biological and Environmental Sciences, University of GothenburgBox 461, 405 30 GothenburgSweden; 2 Institute of Ecology and Earth Sciences, University of TartuLai 40, Tartu 51005Estonia; 3 Department of Organismal Biology, Uppsala UniversityNorbyvägen 18D, 75236 UppsalaSweden; 4 Department of Mathematical Statistics, Chalmers University of Technology412 96 GöteborgSweden; 5 Forest Soils and Biogeochemistry, Swiss Federal Research Institute WSLZuercherstrasse 111, 8903 BirmensdorfSwitzerland; 6 Molecular Ecology, Institute for Sustainability SciencesAgroscope, Reckenholzstrasse 191, 8046 ZurichSwitzerland; 7 Ernst-Moritz-Arndt University, Institute of Botany and Landscape EcologySoldmannstr. 15, D-17487 GreifswaldGermany; 8 Department of Biology, McMaster UniversityHamilton, Ontario, L8S 4K1Canada; 9 Department of Infectious Diseases, Institute of Biomedicine, Sahlgrenska Academy, University of GothenburgGuldhedsgatan 10, SE-413 46 GothenburgSweden; 10 Department of Natural Sciences, The University of FindlayFindlay, OHUSA; 11 Group of Plant Nutrition, Institute of Agricultural Sciences, Department of Environmental Systems ScienceETH Zurich, Eschikon 33, 8315 Lindau (ZH)Switzerland; 12 Natural History MuseumP.O. Box 1172 Blindern, 0318 OsloNorway; 13 Natural History Museum, University of TartuVanemuise 46, Tartu 51014Estonia; 14 Tiburon, CAUSA

**Keywords:** fungi, chimera detection, reference dataset, molecular ecology, PCR artifacts

## Abstract

The nuclear ribosomal internal transcribed spacer (ITS) region is the most commonly chosen genetic marker for the molecular identification of fungi in environmental sequencing and molecular ecology studies. Several analytical issues complicate such efforts, one of which is the formation of chimeric—artificially joined—DNA sequences during PCR amplification or sequence assembly. Several software tools are currently available for chimera detection, but rely to various degrees on the presence of a chimera-free reference dataset for optimal performance. However, no such dataset is available for use with the fungal ITS region. This study introduces a comprehensive, automatically updated reference dataset for fungal ITS sequences based on the UNITE database for the molecular identification of fungi. This dataset supports chimera detection throughout the fungal kingdom and for full-length ITS sequences as well as partial (ITS1 or ITS2 only) datasets. The performance of the dataset on a large set of artificial chimeras was above 99.5%, and we subsequently used the dataset to remove nearly 1,000 compromised fungal ITS sequences from public circulation. The dataset is available at http://unite.ut.ee/repository.php and is subject to web-based third-party curation.

Fungi form a large and diverse group of heterotrophic organisms. Molecular (DNA sequence) data have gradually become a critical research tool in mycology, owing largely to the subterranean or otherwise inconspicuous nature of much of fungal life coupled with a general lack of tangible, discriminatory morphological characteristics in many fungi (12, 33). In many cases, DNA sequences represent the only means for high-precision species identification and delimitation (15). However, molecular mycology is not devoid of complications, and many technical issues and potential pitfalls need to be considered before DNA sequences can be applied for scientific purposes (13, 18). One of these complications is the unintentional generation of chimeric sequences during either PCR amplification or the assembly of individual sequence reads. Chimeras are artificial DNA sequences that are composed of two (or sometimes more) sequence fragments that do not naturally belong together (35). Most chimeras are produced during PCR when DNA templates of more than one sequence type are co-amplified and incomplete amplicons act as primers on not fully matching templates. These template switches are more likely to occur if the targeted gene/marker features a highly conserved segment that is very similar among different taxa in the mixed DNA template pool (9). The resulting chimeric sequence consists of two (or more) parts that originate from different parent sequence types. Chimeras of this kind lack a biological interpretation and need to be removed from any dataset in which they exist. Failure to do so will compromise any analyses the dataset is used in, including species identification and delimitation, richness estimation, and multiple sequence alignment/phylogenetic inference (23).

The nuclear ribosomal internal transcribed spacer (ITS) region is the formal fungal barcode and the most commonly sequenced genetic marker in mycology (2, 28). The average length of the ITS region is 550 base-pairs (bp) in the fungal kingdom, but varies markedly among lineages (8, 29). It is composed of the two variable spacers, ITS1 and ITS2, and the intercalary, highly conserved 5.8S ribosomal gene. The latter readily acts as a bridge-point for chimeric extension in mixed-template PCR, making chimera control an essential part of ITS-based mycological research (31, 32). Studies employing cloning of PCR amplicons need to be particularly vigilant against chimeras. In cloning, a single PCR fragment is selected, multiplied, and sequenced; therefore, any polymerase-generated artifact will penetrate to an extent not observed in direct Sanger sequencing, in which sequence chromatograms represent the averaged signal from numerous original templates rather than a single PCR fragment. Chimera control also has to be exercised when working with individual specimens such as fruiting bodies. Contamination in any of the laboratory steps or the presence of intra-sporocarp parasites or commensals such as *Hyphomycetes* in boletes (4) and lichen-inhabiting lineages in *Tremellales* and *Filboasidiales* (22) may produce chimeras in these cases. Chimera control is also essential in next-generation sequencing of fungal communities in environmental samples, in which the multi-species nature of the samples, sometimes coupled with the intrinsic properties of the sequencing platform, provide ample opportunities for chimera formation (27). Prior to this study, a total of 1,825 chimeras involving public sequences of the fungal ITS region were recorded in the UNITE database for the molecular identification of fungi (1), the largest tailored and actively curated public database for fungal ITS sequences. UNITE mirrors the (Sanger-derived) fungal ITS sequences in the International Nucleotide Sequence Database Collaboration (INSDC: GenBank, EMBL, and DDBJ; 20) such that the chimera count in UNITE is essentially that of the public fungal ITS sequence corpus generated to date by the scientific community.

The detection of chimeras is challenging. Obvious cases of chimeras can be identified in smaller, homogeneous datasets by simply examining the corresponding multiple sequence alignment or using the sequences for a BLAST search in INSDC (Supplemental Fig. S1). Manual approaches become problematic in larger datasets. Nilsson *et al.* (23) released a Perl-based semi-automated chimera finder for fungal ITS sequences, the advantage of which is its ability to detect Sanger-length chimeras occurring at and above the ordinal level; however, it is ineffective against chimeras occurring within the same order. Edgar *et al.* (6) introduced UCHIME, a powerful and feature-rich chimera checker for all major computer platforms and read lengths. In its “reference database mode”, it cleaves all query sequences into four (default) segments in order to determine whether the constituent parts are best matched by different sequences in the reference database; on the UCHIME chimera scale, more obvious mismatches have higher chimera scores. The user is presented with a list of sequences that exceed the chimera score cut-off threshold, and, ideally, need to be examined by hand. UCHIME also offers a “*de novo* mode” of chimera detection for newly generated next-generation sequencing datasets, in which putative chimeras are deduced based on the abundances of the estimated amplicon sequences (denoising used) or unique reads (no denoising used).

A rich and reliable reference database lies at the core of the reference database mode of UCHIME and similar programs. However, such a database is not readily available for fungal ITS sequences. Difficulties have been associated with using the corpus of fungal ITS sequences downloaded from INSDC because this dataset contains a non-trivial number of chimeras and sequences of other technical or annotation-related problems. In the present study, we introduced an incremental, taxonomically inclusive, and high-quality set of fungal ITS sequences (http://unite.ut.ee/repository.php) derived from the INSDC as mirrored in UNITE for use in chimera detection pursuits.

## Materials and Methods

### Compilation of the ITS reference dataset

**Fig. 1:**
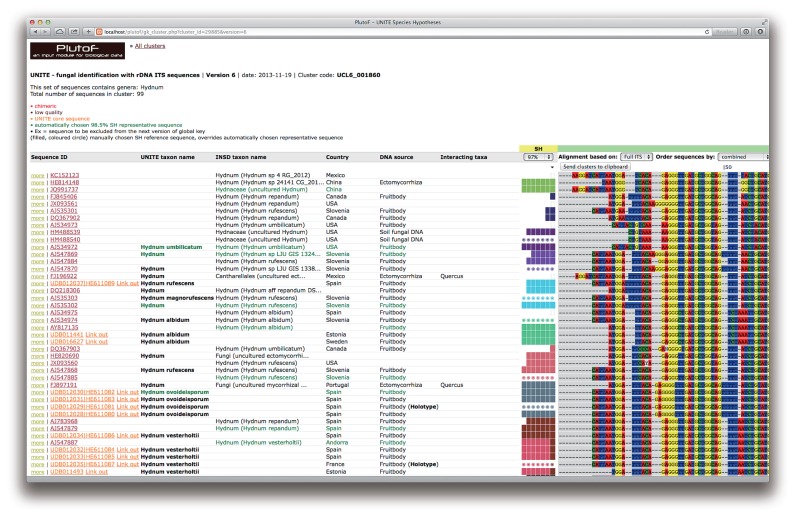
A genus-level alignment in UNITE of the ectomycorrhizal genus *Hydnum*, with the individual species hypotheses (SHs) indicated by the colored boxes at different similarity levels (97–100%). One sequence (shown here in green) from each such species hypothesis was used to build the chimera reference dataset. Manually chosen reference sequences are indicated by filled circles in the SH column; these superseded the automatic choice of representative sequences for species hypotheses and are particularly suited for sequences from type (or otherwise authenticated) material. Two sequences from type specimens are indicated in the figure.

UNITE downloads all fungal ITS sequences from INSDC twice a year, and subjects them to a series of semi-automated quality control measures. All sequences are then clustered at 80% similarity in USEARCH 7 (5) to produce clusters at roughly the genus/subgenus level. A multiple sequence alignment is computed for each such genus-level cluster for graphical display, and the sequences in each cluster are subjected to a second round of clustering, at roughly the species level (97%–100% similarity in 0.5% steps). The resulting operational taxonomic units—called *species hypotheses* (SHs)—are given unique names of the accession number type to enable un- ambiguous communication across studies and datasets, and are reachable through URIs such as http://unite.ut.ee/sh/SH158651.06FU. Although a species hypothesis may be composed of a single sequence if sanctioned manually, the present study focused on species hypotheses consisting of two or more sequences. (As discussed below, many singleton sequences do not meet quality requirements.) When logged into UNITE, the user can view genus-level alignments with the species hypotheses indicated (Fig. 1). Based on the most frequent sequence type in each species hypothesis, a sequence is automatically chosen as a representative sequence (at the 98.5% similarity threshold) for that species hypothesis. These representative sequences serve as the basis for the chimera reference database.

However, additional control may be desired over the sequence chosen to represent a species hypothesis in some cases. Sequences stemming from type specimens, for example, form particularly good candidates to represent a species hypothesis in so far as they are of sufficient length and read quality (17). UNITE offers web-based third-party designation of representative sequences to its users; a user can log in and easily change the choice of representative sequences or the similarity level at which they should be applied. These manually chosen representative sequences are referred to as *reference* sequences. During a recent workshop and subsequent annotation effort (17, 25), the participants, primarily fungal taxonomists, re-selected 2,936 reference sequences and verified another several thousand representative sequences for reliability and representativeness. In addition, 97 sequences that came out as singletons in the 97% clustering step and, hence, did not qualify as species hypotheses were sanctioned as formal species hypotheses by the participants. Schoch *et al.* (29) similarly presented a large number of ITS sequences from type material; these were designated as reference sequences for species hypotheses in UNITE where applicable. The end product was a set of 3,973 reference and 17,086 representative sequences spanning all species hypotheses across the entire fungal tree of life.

### Evaluation of the ITS reference dataset

To estimate the power of the reference dataset to detect chimeras under ideal (artificial) conditions, we manipulated the 21,059-sequence reference dataset to contain only chimeric sequences; all sequences were bisected in the middle of the 5.8S gene, and the fragments were reshuffled randomly to produce a total of 21,059 chimeras. Each of these consisted of ITS1 + half of the 5.8S gene from one sequence, and the remainder of the 5.8S gene + ITS2 from another. Fragments were not allowed to graft back onto their parent sequence. The procedure was repeated ten times to produce ten different (21,059-sequence) datasets of chimeras. We ran these new chimeras through UCHIME using the present reference sequence file as the reference corpus.

### Chimeras among public ITS sequences

In an effort to detect and flag the worst cases of chimeric fungal ITS sequences in INSDC/UNITE, we ran the combined INSDC/ UNITE dataset (376,840 more or less full-length fungal ITS sequences, excluding the 1,825 known chimeras) as a query in UCHIME 7.0.1090 using the default chimera score cut-off value (0.28) and the new 21,059-sequence dataset as the chimera-free reference dataset. We examined the 5,414 resulting putative chimeras manually for a chimeric nature following the procedure of Nilsson *et al.* (24). In order to evaluate the performance of UCHIME on these authentic sequences, we compared UCHIME scores for the sequences we deemed to be chimeric with the scores for those that were not considered to be chimeric by us. The Wilcoxon-Mann- Whitney test implemented in R 2.15.3 (http://cran.r-project.org) was used to statistically analyze differences in scores between the chimeric and non-chimeric sequences.

## Results

### Reference dataset

The reference dataset comprised 21,059 sequences (August 2014) and is available for download at http://unite.ut.ee/repository.php. It was designed for chimera control of more or less full-length fungal ITS sequences, and may, thus, not work well for sequences that contain non-trivial parts (200+ bp.) of the neighboring small-subunit (SSU/18S) and/or large subunit (LSU/28S) genes because the SSU and LSU are absent from the reference dataset. We also provide standalone ITS1- only and ITS2-only files extracted using ITSx for the same 21,059 sequences (3). This supports chimera detection in datasets containing only (full-length or partial) ITS1 or ITS2 sequences, notably those stemming from amplicon-based next-generation sequencing efforts.

### Evaluation of the ITS reference dataset

UCHIME identified an average of 99.82% (SD 0.0421) of the sequences in the ten 21,059-chimera datasets as chimeric by using default UCHIME settings.

### Chimeras among public ITS sequences

**Fig. 2:**
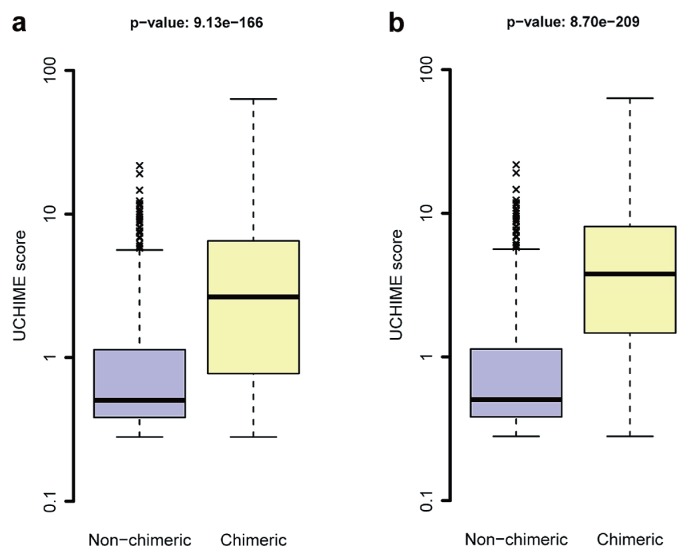
Boxplots of UCHIME scores for non-chimeric and chimeric sequences. All sequences were included in panel (a), while sequences with low read quality were removed in panel (b). The score difference between the non-chimeric and chimeric sequences was statistically assessed through the Wilcoxon-Mann-Whitney test.

A total of 5,414 (1.4%) out of the 376,840 UNITE/INSDC sequences were identified as putatively chimeric by UCHIME, ranging from cases with very high chimera scores (50+) to those barely exceeding the threshold value (0.28; Fig. 2). One hundred and eighty-seven sequences had a chimera score above 10; 768 had a chimera score between 1 and 10; and 4,459 sequences had a chimera score above the 0.28 threshold, but below 1. All these sequences were subjected to a manual examination in the INSDC and UNITE. We identified 724 (13.3%) as clear cases of chimeras and 239 (4.4%) that represented sequences of low read quality; however, we could not reach an unequivocal decision on the chimeric nature of the remaining 4,551 entries or a chimeric nature appeared to be unlikely. Screenshots of the BLAST results of these entries are shown in Supplemental Fig. S1. Approximately 25% of these sequences appeared to stem from fungi with conserved ITS2 regions, but variable ITS1 regions (*e.g.* lineages in *Aspergillus* and *Colletotrichum*; see 17), and approximately the same percentage of sequences appeared to be natural hybrids (cf. 10, 26), as evidenced by the multiple independent recoveries of many of these species. Although 10% of the sequences appeared to be of low read quality, explicit evidence was lacking. A decision on whether the remaining sequences were chimeric was dependent on one to five base-pairs. We concluded that unequivocal decisions were impossible in these cases, and the sequences were left in the database. Ten cases of incorrectly assembled sequences (“assembly chimeras”) were also detected. Clear cases of chimeras, the assembly chimeras, and low-read quality entries were marked as compromised in UNITE and reported to the INSDC.

The Wilcoxon-Mann-Whitney test revealed significant differences in scores between sequences considered chimeric and those that were not found to be chimeric, with median UCHIME scores of 3.78 and 0.51 for the chimeric and nonchimeric groups, respectively (*p*<10^−16^; Fig. 2).

### Database implementation

We modified UNITE to hold and display UCHIME results in order to facilitate chimera detection for users with limited experience in command-line programs. Sequences that exceeded the default score at which UCHIME considered a sequence chimeric have now been marked as putatively chimeric in the database, with the score and two putative parent sequences indicated and hyperlinked (Supplemental Fig. S2). These data are re-generated after each re-computation of the species hypotheses in UNITE. The 5,414 sequences indicated as putatively chimeric in UNITE/INSDC in the present study were specified accordingly.

## Discussion

We here present a 21,059-sequence fungal ITS dataset for use in chimera control in UCHIME or any other chimera detection program that relies on a chimera-free reference dataset. Amplicon-based next-generation sequencing studies are strongly advised to consider the reference-free *de novo* chimera detection mode of UCHIME/UPARSE (7); however, even in these studies, an established reference dataset may need to be referred to, at least for particularly problematic cases. Our sequence dataset, which is available at http://unite.ut.ee/repository.php, is updated automatically as the number of fungal ITS sequences in INSDC increases. It is furthermore subject to third-party annotation, such that those who feel they are in a position to improve the data (and particularly the choice or similarity levels of applications of reference sequences for species hypotheses) can do so. Nevertheless, it is not a dataset devoid of potential problems, with the most obvious one being its limited taxonomic depth. Although the ITS region is the formal fungal barcode, ITS sequences are only available for ~17,000 fully identified species out of the estimated one to several million extant species of fungi (11, 17). Chimera detection programs, in contrast, work better when both parent sequences of a chimera are present in the reference database. This will, in practice, not always be the case for those processing newly generated ITS sequences from environmental or taxonomic studies, suggesting that a certain proportion of false-positive (and false-negative) identifications will have to be tolerated by the mycological community for now. This testifies to the importance of manual verification of sequences indicated to be putatively chimeric (and possibly also sequences that are close to, but do exceed the threshold for what is regarded a chimeric sequence).

A second shortcoming lies in the requirement that a species hypothesis in UNITE must be composed of two or more sequences (although singleton sequences can be sanctioned manually as species hypotheses). This requirement is important from a sequence quality point of view because sequences that form singletons in clustering approaches of large datasets are often associated with quality problems (13, 24, 30). To automatically endow all singleton sequences with the status of species hypotheses may give rise to a large number of phantom species hypotheses. Such species hypotheses may not correspond to any biological reality because the sequences are in some way technically compromised. The inclusion of these sequences in the chimera reference dataset may reduce the power of the dataset for chimera detection. Nevertheless, a certain proportion of singleton sequences do correspond to high-quality DNA sequences that, although not finding any close match in the sequence databases, do correspond to actual species. These have, by default, not been included in the present reference database. UNITE provides the opportunity to manually sanction such singleton sequences as reference sequences for species hypotheses. Manual examinations and designations form a bottleneck here, and the absolute majority of reference sequences that have been designated in UNITE are found in species hypotheses composed of two or more sequences. This number is expected to rise because the number of third-party annotators is increasing (25). The requirement that a species hypothesis must be composed of two or more sequences excludes most spurious sequences from being used as representative sequences, but does not offer full protection against unwanted representative sequences. For example, the exact same chimeric sequence may be formed twice or even more in the same study or across studies. Therefore, our “chimera-free” reference dataset may still contain a few chimeras. Smaller chimeric insertions (or untrimmed vectors) in sequences, in which the insertions are small enough not to penetrate to give rise to different species hypotheses, may also be present in the dataset. Although we are not currently aware of such a case, we ask the users to be aware of their potential existence in the dataset and to take action when they find any such entries. A final shortcoming lies in the fact that ITS chimeras spanning kingdoms, such as Fungi and Plantae, are, although unlikely, at least conceivable (21). The extent to which the present reference dataset, which covers fungi only, can be used to find cross-kingdom chimeras has yet to be established.

The intrinsic properties of the ITS region may also make successful chimera detection difficult. The highly variable ITS1 and ITS2 subregions may differ slightly in variability, with the length and sequence content of ITS2 being more conserved, at least in *Basidiomycota* (14, 34). An ITS2 sequence type may be conserved across several species whereas the corresponding ITS1 sequences differ in length/ sequence content by a few base-pairs or more. These cases may give rise to false-positive chimera detections in which ITS1, due to its being unique, will be assigned to the correct parent sequence A, whereas ITS2 will be assigned by chance to parent sequence B. This designation occurs due to sequences A and B having identical ITS2 sequences. The multicopy nature of the ITS region, with the potential for several different allelic variants of the marker, may occasionally give rise to chimeras that may be both difficult to find and complicated to verify (19). A few cases of naturally occurring “chimeras” have been reported previously (16, 36). To summarize, the user needs to be aware that it is not always possible to unequivocally prove that a sequence is chimeric. This is particularly true for sequences downloaded from public sequence databases, in which the context of the sequence, as well as additional, explanatory data, will not always be available for examination.

We demonstrated that the UCHIME/reference dataset combination was useful in the pursuit of chimeric fungal ITS sequences in the public corpus; nearly 1,000 substandard publicly deposited sequences were identified and removed. Nevertheless, we do not want to imply that fungal ITS chimera detection will be trivial from this point on. When we manipulated the reference dataset to contain only chimeric sequences, the UCHIME/reference dataset combination detected an average of 99.82% out of the 21,059 chimeras as chimeric, which was considered to be a very satisfactory performance. However, this approach relied on the random regrafting of sequence halves, which, in reality, may be more common among closely related species (similar sequences). We similarly assumed chimeric breakpoints to occur in the very conserved 5.8S gene, which may be an oversimplification. Thus, the user should not expect a chimera detection efficiency of 99.82% in real-life datasets. Nevertheless, we believe that the present dataset will lead to noise reduction in fungal ITS datasets in medical, taxonomic, and environmental sequencing of fungi and fungal communities. We also hope that users will report any chimeric sequences detected in INSDC/UNITE to the database to prevent bad data from propagating through the literature. We invite the community to improve the present dataset through UNITE, particularly by designating reference sequences for species hypotheses and excluding substandard entries. Other improvements, such as taxonomic re-annotations, are also valuable to the mycological community. The time dedicated to third-party annotation is arguably a small price to pay for the knowledge that chimera detection and molecular identification will be performed in a richer and more informed way by the user and the remainder of the scientific community.

## Supplementary Information
