# Supplementary material for: A Comprehensive, Automatically Updated Fungal ITS Sequence Dataset for Reference-Based Chimera Control in Environmental Sequencing Efforts
Source: Microbes Environ. 2015 Mar 19;30(2):145–50. doi: 10.1264/jsme2.ME14121 (PMC4462924; doi:10.1264/jsme2.ME14121)

This document contains Figs. S1 and S2 of Nilsson et al., "*A comprehensive, automatically updated fungal ITS sequence dataset for reference-based chimera control in environmental sequencing efforts*".

Fig. S1 (page below). Graphical illustration of problematic sequences with further explanations on how to manually identify compromised sequences.

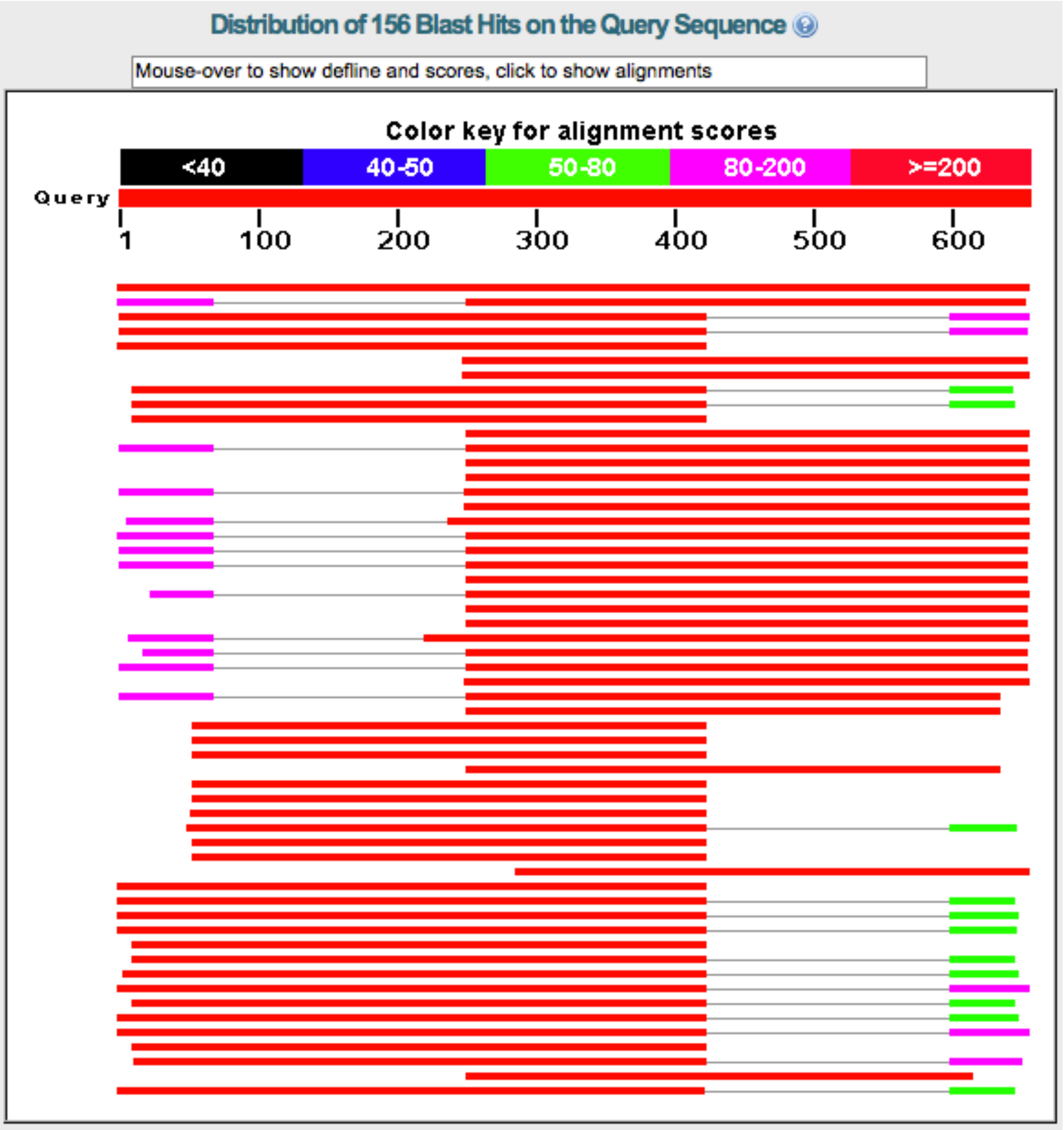

Figure S1-1a. Graphical BLAST overview in GenBank of a chimera (JQ272336). The chimera itself was used as query sequence, such that it is both query sequence and its own best BLAST match. The remaining sequences show a very alarming pattern: alternation of matching regions. Either the ITS1-5.8S part matches the query sequence well, or the 5.8S-ITS2 part matches the query sequence well. No non-self GenBank sequence matches the query sequence over its full length. This is never a good sign and should always be taken to mean that manual examination is necessary.

```
Range 1: 1 to 414 GenBank  Graphics                     ▼ Next Match  ▲ Previous

Score             Expect   Identities       Gaps          Strand
682 bits(369)     0.0      399/414(96%)     0/414(0%)     Plus/Plus

Query  11   TTAGAGGAAGTAAAAGTCGTAACAAGGTTTCCGTAGGTGAACCTGCGGAAGGATCATTAT  70
            ||||||||||||||||||||||||||||||||||||||||||||||||||||||||||||
Sbjct  1    TTAGAGGAAGTAAAAGTCGTAACAAGGTTTCCGTAGGTGAACCTGCGGAAGGATCATTAT  60

Query  71   CGTACAACAGGAGGTGCCGAGGTTATCGCTGACCCTAGGTCGTGCACGCCTCGAGCATTC  130
            |||||||||||||||||||||||| |||||||||||||||||||||||||||||||||||
Sbjct  61   CGTACAACAGGAGGTGCCGAGGTTGTCGCTGACCCTAGGTCGTGCACGCCTCGAGCATTC  120

Query  131  TTACTCCTCATCCACTTCACCCCTTGTGCATCCCCGCGTGGGCTCTCTCGAAAGAGAGAG  190
            ||||||||||||||||||||||||||||||||||||||||||||||||||||||||||||
Sbjct  121  TTACTCCTCATCCACTTCACCCCTTGTGCATCCCCGCGTGGGCTCTCTCGAAAGAGAGAG  180

Query  191  CCGGCGTCATTACACAAACACAAAGTAGCAGTAGAATGTTCTTTGCATTTGTATGCAGTT  250
            ||||||||||||||||||||||||||||||||||||||||||||||||||||||||||||
Sbjct  181  CCGGCGTCATTACACAAACACAAAGTAGCAGTAGAATGTTCTTTGCATTTGTATGCAGTT  240

Query  251  AATACAACTTTCAGCAACGGATCTCTTGGCTCTCGCATCGATGAAGAACGCAGCGAAATG  310
            ||||||||||||| |||||||||||||||||||||||||||||||| |||||||||||||
Sbjct  241  AATACAACTTTCAACAACGGATCTCTTGGCTCTCGCATCGATGAAGGACGCAGCGAAATG  300

Query  311  CGATAAGTAATGTGAATTGCAGAATTCAGTGAATCATCGAATCTTTGAACGCACCTTGCA  370
            ||||| ||||||||||||||||||||||||||||||||||||||||||||||||||||| 
Sbjct  301  CGATACGTAATGTGAATTGCAGAATTCAGTGAATCATCGAATCTTTGAACGCACCTTGCG  360

Query  371  CTCCTTGGTATTCCGAGGAGTATGCCTGTTTGAGTGTCATGAAATCATCAACCT  424
            | |||||||||||||| | | |  || |||||||||||||||| |  |||||||
Sbjct  361  CCCCTTGGTATTCCGAAGGGCACACCCGTTTGAGTGTCATGAAGTTCTCAACCT  414
```

Figure S1-1b. Alternation of matching regions as seen from the BLAST alignments (same accession number). Here, it is the ITS2 region that does not match the query sequence well, whereas the first part of the sequence is aligned well over more than 400 bp.

```
Range 1: 175 to 583 GenBank  Graphics              ▼ Next Match  ▲ Previous Match

Score             Expect   Identities       Gaps          Strand
688 bits(372)     0.0      398/410(97%)     3/410(0%)     Plus/Plus

Query  249  TTAATACAACTTTCAGCAACGGATCTCTTGGCTCTCGCATCGATGAAGAACGCAGCGAAA  308
            ||||||||||||||||||||||||||||||||||||||||||||||||||||||||||||
Sbjct  175  TTAATACAACTTTCAGCAACGGATCTCTTGGCTCTCGCATCGATGAAGAACGCAGCGAAA  234

Query  309  TGCGATAAGTAATGTGAATTGCAGAATTCAGTGAATCATCGAATCTTTGAACGCACCTTG  368
            ||||||||||||||||||||||||||||||||||||||||||||||||||||||||||||
Sbjct  235  TGCGATAAGTAATGTGAATTGCAGAATTCAGTGAATCATCGAATCTTTGAACGCACCTTG  294

Query  369  CACTCCTTGGTATTCCGAGGAGTATGCCTGTTTGAGTGTCATGAAATCATCAACCTCATA  428
            |||||||||| ||||||||||||||||||||||||||||||||||||  |||||||||||
Sbjct  295  CACTCCTTGGCATTCCGAGGAGTATGCCTGTTTGAGTGTCATGAAATTCTCAACCTCATA  354

Query  429  GTTTTATTACCC-TGAGGCTTGGACTTGGAAGCTGCTGGCTTATGTCGGCTCTTCTTAAA  487
            |||||||||| | ||||||||||| |||||||||||||||| ||||||||||||||||||
Sbjct  355  GTTTTATTACTCTTGAGGCTTGGATTTGGAAGCTGCTGGCTAATGTCGGCTCTTCTTAAA  414

Query  488  TATATTAGCTGCTTGTCCGTGAGTTGGCTCAGAGTGTGATAAGTATCTACACTTGGTGGC  547
            | ||||||||||||||||||||||||||||||||||||||||||||||||||||||||||
Sbjct  415  TGTATTAGCTGCTTGTCCGTGAGTTGGCTCAGAGTGTGATAAGTATCTACACTTGGTGGC  474

Query  548  TGCTCGCATTGGATGTGGCTTCTAAT-CGTCTTCGGACAATTTTTTAAGATCTGACCTCA  606
            |||| ||||||||||||||||||||| |||||||||||||  ||||||||||||||||||
Sbjct  475  TGCTTGCATTGGATGTGGCTTCTAATCCGTCTTCGGACAAC-TTTTAAGATCTGACCTCA  533

Query  607  AATCAGGTAGGACTACCCGCTGAACTTAAGCATATCAATAAGCGGAGGAA  656
            ||||||||||||||||||||||||||||||||||||||||||||||||||
Sbjct  534  AATCAGGTAGGACTACCCGCTGAACTTAAGCATATCAATAAGCGGAGGAA  583
```

Figure S1-1c. A few entries down the list (same accession number), it is the ITS1 of the query sequence that does not match the reference sequence well (no

alignment produced between the query and the subject for the first few hundred base-pairs). This bespeaks a 5.8S-based chimeric union. We recommend examining the BLAST alignment to learn of the specifics of each putative chimera. In cases where the graphical summary is not as clear-cut as above (and indeed when the graphical summary does not indicate any problem at all), the BLAST alignment is the best source for further examination.

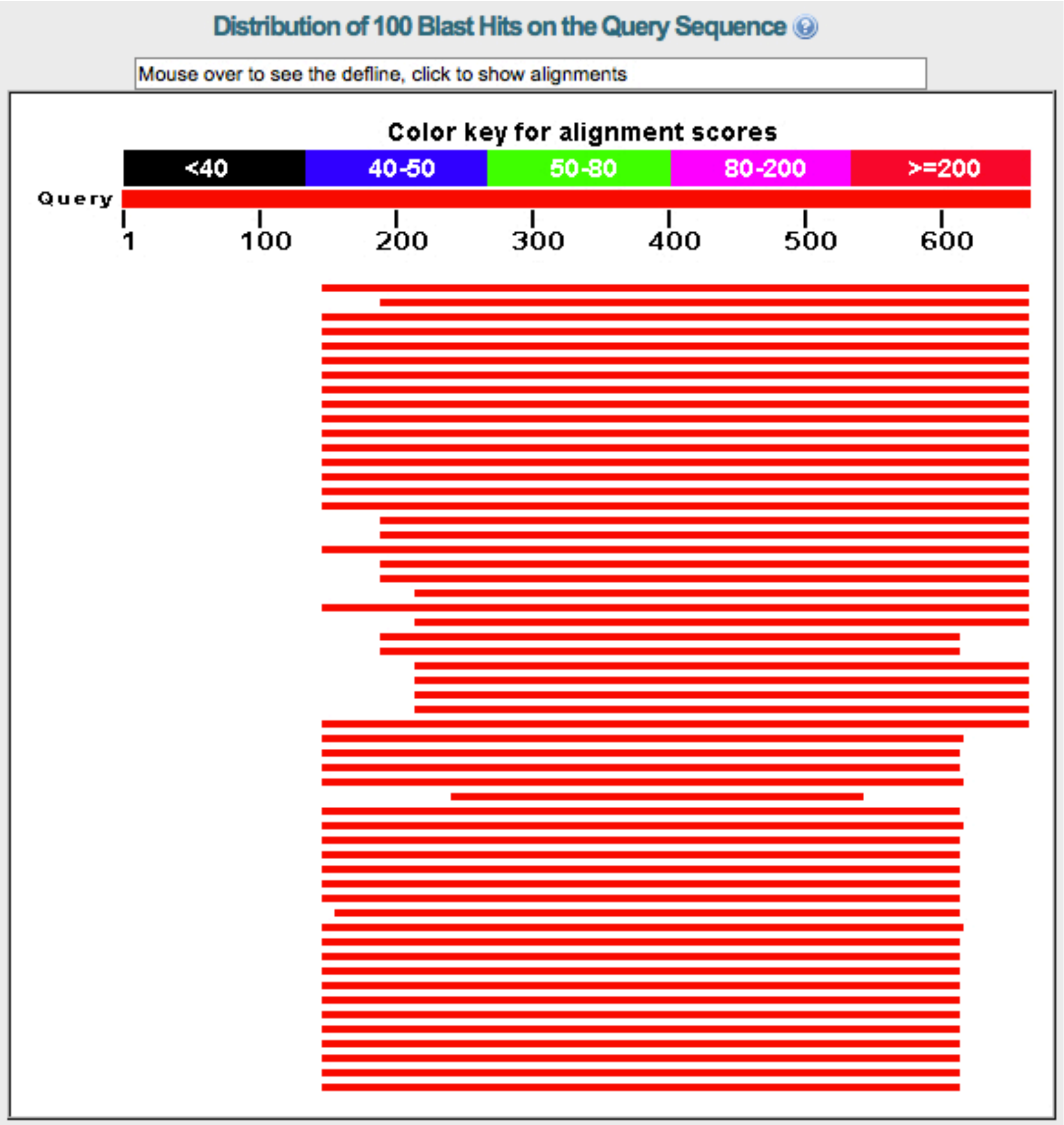

Figure S1-2. Entry JN035638 shows a very alarming pattern: a complete lack of matches for the first part of the query sequence. This is not to be expected for a high-quality fungal ITS sequence, and manual examination of the BLAST results are always needed in these cases. BLAST:ing the first part of the sequence separately is one way forward, as is examining the first part of the query sequence for indications of low read quality.

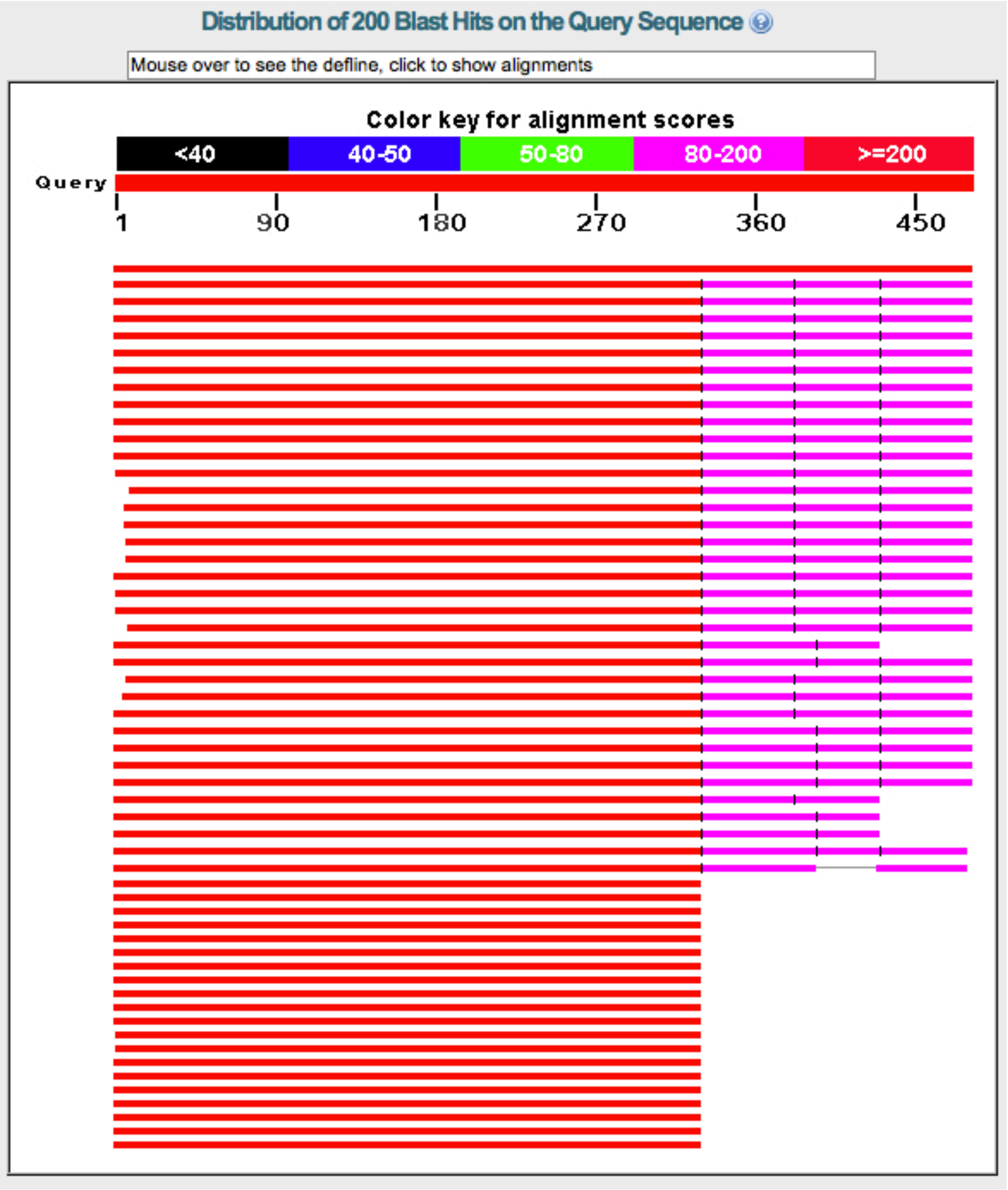

Figure S1-3. Entry FM180040 shows another alarming pattern: breaks in the BLAST alignment. This typically means that the BLAST results cannot be shown in a straightforward way, such as when a sequence was assembled incorrectly. Assembly chimeras produce BLAST results like this.

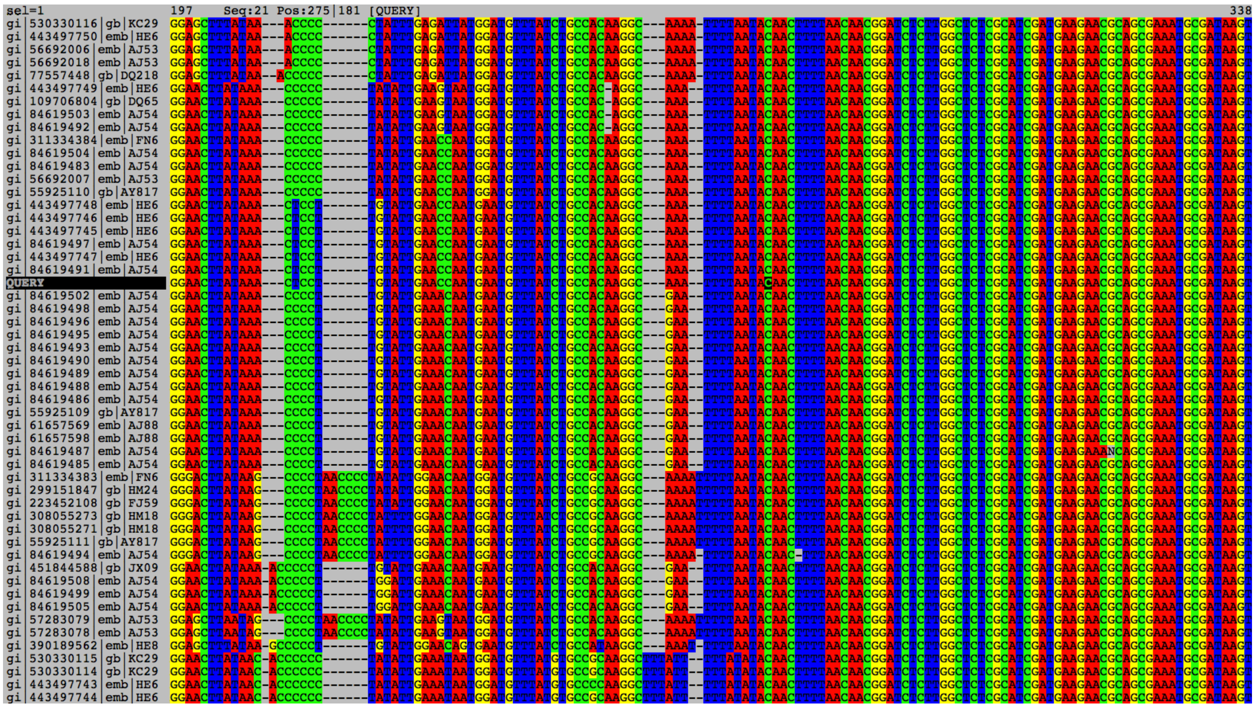

Figure S1-4a. The sequence QUERY (highlighted) is chimeric. The sequence aligns perfectly in the ITS1 and the first part of the 5.8S – the screenshot shows the last part of the ITS1 and the first part of the 5.8S. The cursor approximately in the middle of the screen indicates the first base-pair of the 5.8S. The sequences come from the genus *Hydnum*.

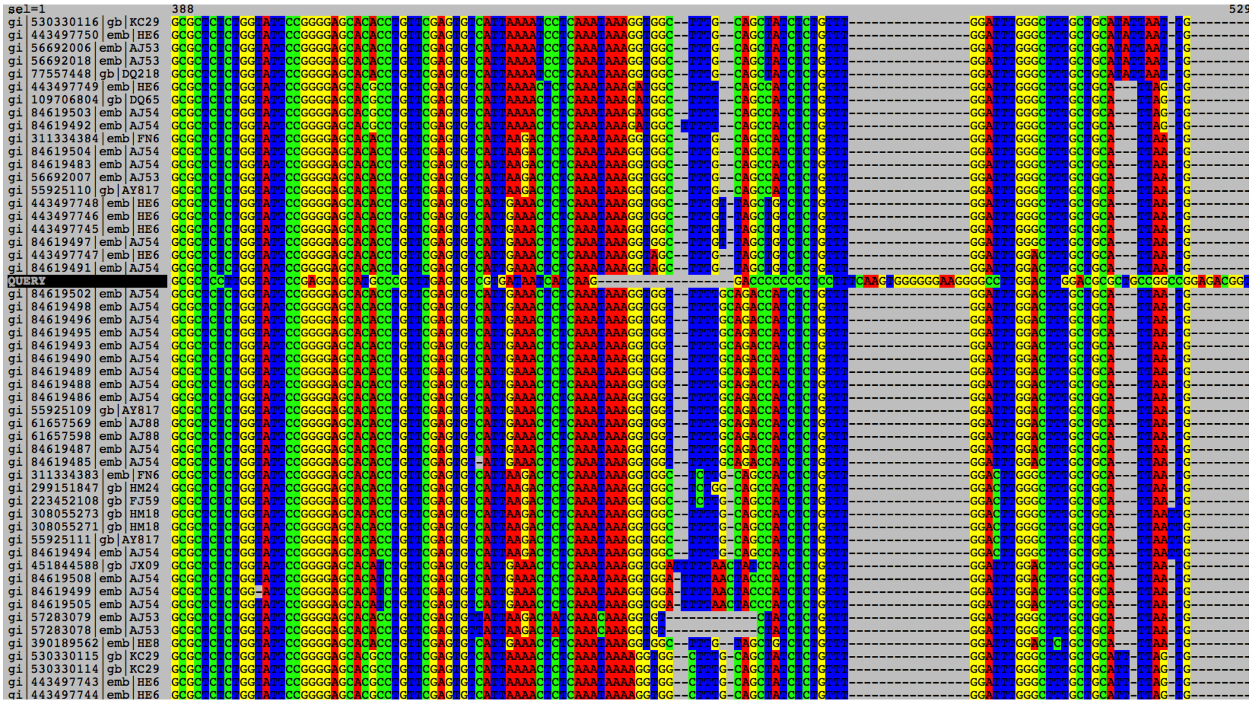

Figure S1-4b. However, as one scrolls through the 5.8S gene, the chimeric nature of the entry QUERY becomes evident. This screenshot shows the last base-pairs of the 5.8S and the start of ITS2. A separate BLAST search of the ITS2

shows it to belong to a *Botryobasidium* species (same order as *Hydnum,* but a different family). The alignment was computed in MAFFT and displayed in SeaView.

ITS Primer Maps – ITS primer maps are available at

https://unite.ut.ee/primers.php,
http://sites.biology.duke.edu/fungi/mycolab/primers.htm, and
http://lutzonilab.org/nuclear-ribosomal-dna/

Fig. S2. Screenshot showing how potentially chimeric sequences are indicated in UNITE through the use of UCHIME.

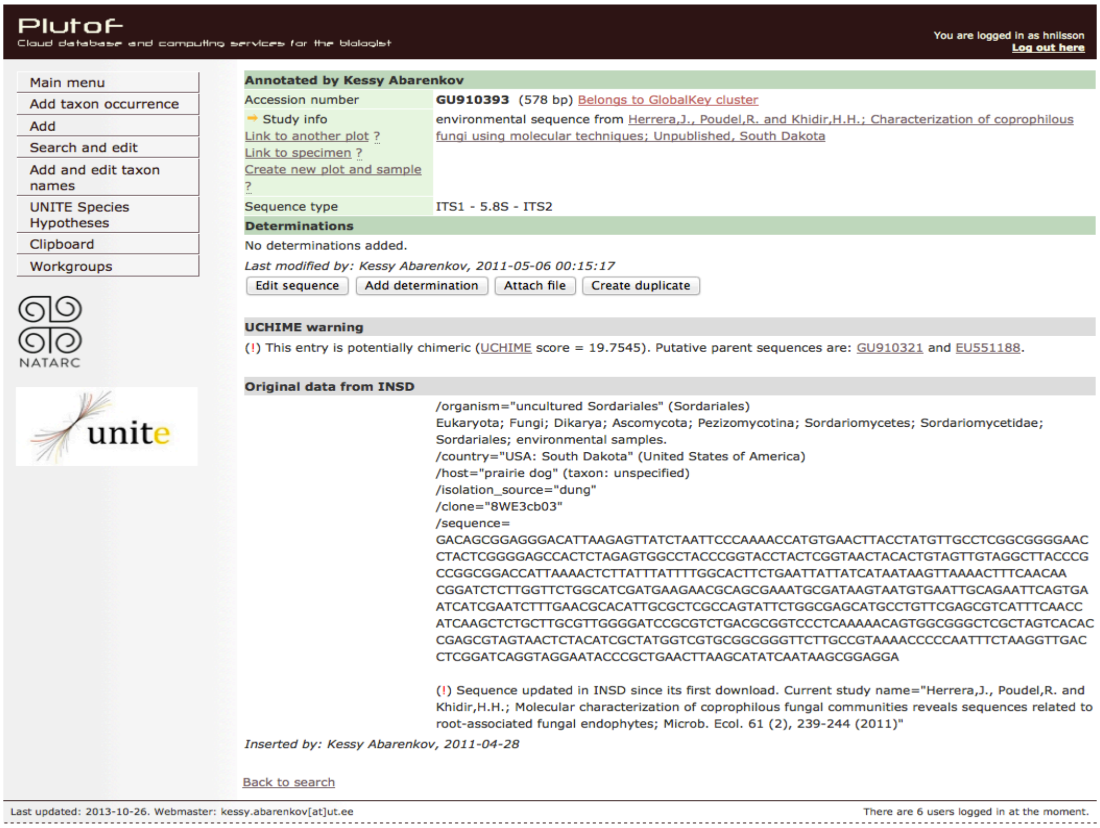

Supplement: Supplementary file 1 [file 30_145_s1.pdf]
